# Holding pediatric cardiac surgery together: My Serbian experience of 25 years

**DOI:** 10.3389/fped.2022.800633

**Published:** 2022-09-21

**Authors:** Mila Stajević

**Affiliations:** ^1^Medical School, University of Belgrade, Belgrade, Serbia; ^2^Department of Pediatric Cardiothoracic Surgery, Mother and Child Health Institute of Serbia “Dr Vukan Čupić”, Belgrade, Serbia

**Keywords:** pediatric cardiac surgery, Serbia, 25 years, Mother and Child Institute, Belgrade

## Introduction

It was during my final year at the medical school in Belgrade, Yugoslavia, that I first came into contact with congenital heart surgery (CHS). My initial interest was general pediatric surgery, but due to different circumstances, I had converted to pediatric cardiac surgery after 2 years of basic surgical training. One of the compelling reasons was an opportunity to train abroad due to existing contacts with The Hospital for Sick Children, Great Ormond Street Hospital (GOSH) in London—a non-existing option in other departments.

The Department of Pediatric Cardiac Surgery was formed as a separate department in 1982 at the Mother and Child Health Institute of Serbia (MCHI), a tertiary pediatric center and university teaching hospital. Prior to this, congenital heart surgery had been performed by adult cardiac surgeons, but there are no reliable data on the volume of cases and results. The dedication and ambition of the staff of the new department quickly evolved into an exclusive service where beside the arterial switch operation (ASO), the Fontan operations were introduced at the same time as in the Western world (1).

The disintegration of Yugoslavia started in the early 1990's of the 20th century, significantly affecting expensive and demanding pediatric medicine such as congenital heart surgery. The number of surgeries decreased, palliation took over open-heart cases, and most of the patients were not even able to reach medical facilities.

This article is written as a personal and one-sided view of pediatric cardiac surgery in Serbia. There are several reasons for this approach when reporting the subject. I have been positioned as the Head of Department of pediatric cardiac surgery in my hospital for 24 years, the department being founded 40 years ago. The only preserved written information about these first 15 years are in the form of three operative and intensive care protocol books, scarcely available patient histories, and no computer data whatsoever. My predecessors are either deceased or have been retired or are working in other institutions. My current department colleagues are young and cannot recall circumstances before 2010 (2).

## My capacity in cardiac surgery

**Figure 1:**
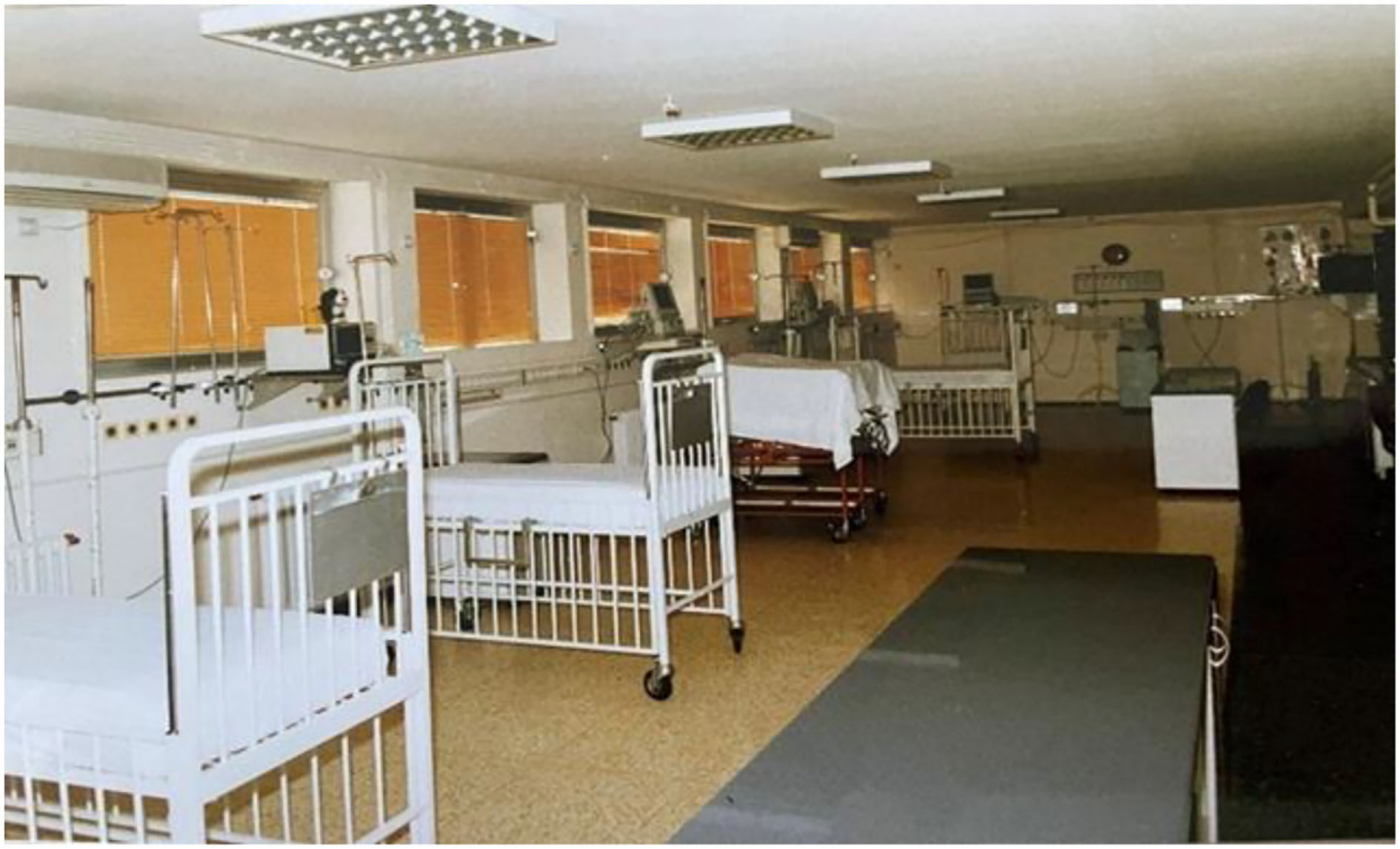
CICU in 1997/1998. Monitoring systems provided by ICHFNO.

I graduated from medical school in 1986 and started my general pediatric surgery residence in 1988, but for reasons mentioned before, I joined the cardiac team in January 1990. The pediatric medicine was probably on its lowest level in 1991. The entire hospital of 400 beds had three or four ventilators and approximately 10 or 15 syringe pumps. As a part of the previous connections with GOSH in London, United Kingdom, I had applied and was appointed as a very young cardiac surgery trainee for the senior house officer position at GOSH in 1992 for 12 months, Mr Jaroslav Stark, a well-known pediatric cardiac surgeon, being my mentor. My contract was extended for additional 6 months (Figure 1).

During my training in London, in the war-stricken Serbia, people were living in one of the worst hyperinflations known in the modern economic history, a humanitarian foundation “For the Child's Heart” was established in Belgrade in 1992 with a “sister” foundation in The Netherlands, by a group of medical and financial professionals as an attempt to help children with CHD. There were more than 400 children on the waiting lists to be operated (3). Paradoxically, a number of patients were sent abroad for cardiac surgery; the price of one intervention abroad was equal to the price of 25 surgeries at home. The foundation financed and supplied the department of pediatric cardiac surgery in those war-stricken years and has continued to do so today, although to a lesser extent (4).

This type of financing pediatric cardiac surgery could not last for long. Therefore, the standard and quality of the pediatric cardiac surgery service were uneven, and palliative procedures dominated corrective surgery. While the CHD surgery in Europe flourished, this European country was performing the surgery of the late 1970's. Although the situation at home had not improved, I had no doubts about returning to Belgrade. In 1994, I had qualified in pediatric surgery and continued to work in my basic department. I started operating on simple cases under supervision and spent a lot of time in the intensive care.

Personal and professional conflicts became apparent at the time; the team members were dissatisfied with the treatment the hospital administration paid to cardiac surgery. I was dissatisfied with the treatment I was given, taking numerous on-calls in the cardiac intensive care unit (CICU), not being able to operate, and spending time typing. My attempts to introduce things I had learnt in London resulted often in unpleasant discussions.

The following conversation was paradigmatic within the department in 1995.

“*Young lady, cardiac surgery has not started with you taking interest in it...”*

*I agree, “was my answer, “but it hasn't relinquished, either, with you losing interest in it”*

Moreover, there was another pediatric hospital in Belgrade with an excellent cardiology team and favorable, lucrative political connections at the time. They promoted the idea of creating a new pediatric cardiac center just a few miles from the existing hospital where I worked and in the ongoing decade of poverty and economic sanctions. Torn between despair about my future career and the loyalty to the hospital I loved, and the fact that I had not been planned to be a part of the future team, I wrote a letter to Mr Jaroslav Stark, asking him to obtain a new training position in the United Kingdom. Again, the golden chariot of opportunity stopped at my door. I was the second person from Central and Eastern Europe who was appointed at GOSH as a senior registrar. To be honest and looking from this perspective, that is coming from Serbia, I was an amateur compared to my two colleagues with whom I had spent that year and who came from well-known American centers. My first 6 months and my efforts to catch up with them and meet the demands of the position were exhausting. I am convinced I kept my position only due to the traditional British patience, their educational skills, my hardwork, and the fact that the consultants knew me from my previous stay. The second half of the year was better; I had grown more confident, my skills improved, and I left for home proud of myself having managed to complete this training.

Upon arrival to Belgrade, I was offered a position as the head of the department. Half of the team was waiting to move to the newly created center, although everything was delayed. The most senior surgeon, my previous chief, had decided to stay. I took the position and became superior to the surgeons who were my senior colleagues. This had created impossible situations in many ways. To make matters more complicated, I got married and pregnant but continued to work until the end of my pregnancy and started working again when my child was 3 months old. It may not seem strange for others, but in this country, it is exceptionally rare to take only a 4-month maternity leave.

## Pediatric cardiac surgery and the Millenium

### Pediatric cardiac surgery and international children's heart foundation

At about the same time in 1997, my hospital was approached by the International Children's Heart Foundation (ICHFNO) led by Dr. William Novick. It was rumored I had invited Dr. Novick to Belgrade, but I had no knowledge of this foundation. A project with the Ministry of Health and the Health Insurance was initiated at the end of 1997. Dr. Novick had previous experience with working in the region (Croatia). From my point of view, the engagement of the ICHFNO completely changed the concept of pediatric cardiac surgery in many ways. We started cardiac surgery rarely or never been done before, that is, complex redo surgery, the Fontan operation, the total cavopulmonary connection (TCPC), the complex left ventricular outflow tract obstructions (LVOTO), the arterial switch operation, and the Senning operation on older babies with D-TGA. Some of these techniques were previously done in the early 1980's but faded off due to the dissolution of the country. This program had been based on the intermittent visit model, with three to four visits per year, and lasted until July 2001 when it was abruptly ended. A total number of 89 children were operated. I was never given an official explanation about this move (5).

### The 21st century

Beside myself, my senior colleague and former Chief had stayed at our hospital. After a personal tragedy, he had changed his attitude of life and let alone cardiac surgery. Still, he was there as a valuable part of the team and supported until 2001. A young, talented doctor, 8 years junior to me joined in 1998. He was a quick learner, with a capacity to look at the world from a different perspective from our country had offered in the 1990s. Cardiac surgery pushed him through the hard and endless work, but with rewards, no other specialty could offer in our hospital (training abroad, conferences, the privilege of meeting, and knowing the “aristocracy” of our surgery). He had also spent a year at GOSH. He left our hospital in 2018, and I am content that we had spent 20 years in more or less happy surgical union; I know it has not always been easy to keep up with my aggressive drive, I had always thought he could do more, his occasional phlegmatic attitude would infuriate me, but he had contributed with many innovations to our work.

Another young surgeon joined us in 2002. After completing his year at GOSH as our trainee (the fifth surgeon from our hospital who had the privilege to be a part of this unique training), he left for the another hospital in 2009. He decided that he had a better surgical future elsewhere. This, of course, added a new work overload on me and the rest of the team.

My team was on the brink of falling apart or fatally cracking a couple of more times.

One of the most soothing lectures to my ears which proved to be true was a presentation given by Mr Frank Molloy, clinical nurse specialist at the ICHFNO and advanced nurse practitioner (GOSH) at the 6th World Conference of Pediatric Cardiology and Cardiac Surgery in Cape Town, RSA, in 2013. The conclusion of the lecture was that in spite of people leaving, if the program is good and opportunities offered, there would be always someone who would be attracted to pediatric cardiac surgery. The existence of my department today strongly supports this statement (6).

This policy brought to me an extremely hardworking and intelligent young surgeon in 2011 who was given a lot of responsibilities prematurely, and although there is a significant age gap between us, I am grateful to have him. His enthusiasm has also had a positive impact on his peers, so now we have another talented young cardiac surgery resident.

Currently, our department has six fully equipped bed spaces in the CICU and 12 beds in the ward where preoperative and postoperative patients are followed (7). Pediatric thoracic surgery is also a part of our everyday surgical routine.

#### Pediatric cardiology

My hospital has a long-standing, half-a-century-long history of pediatric cardiology. I am not going to reflect on the pioneering fantastic work of my colleagues as it would take many pages. The relationship of a pediatric cardiac surgeon and the pediatric cardiologist has been for me one of the most challenging, disturbing, and, at the same time, gratifying interpersonal relationship existing between two physicians. The beginning of my independent surgical career under the circumstances was dramatic. I had two senior, very experienced cardiologists facing me who were professionally adapted and accustomed to my senior departing colleagues. There was one cardiologist of my age though. Beside my relative surgical incompleteness, there were many very risky and, I dare say, inoperable patients who had paid the price of the overdue surgery. Parents were familiar with the cardiologists but still unfamiliar with the young local surgeon. They expected miracles with the arrival of “foreign surgeons.” With the ICHFNO program commencing, I had delayed most of the complex operations for the American visits. There were many dramatic situations, deceased children, disappointments, and, fortunately, positive surprises. I was often alone when postoperative deaths were to be explained to grieving parents. However, with time, as my experience and self-confidence grew, the gap between the senior cardiologists and us in cardiac surgery slowly diminished. The next highly inflammatory situation arose when we had decided to start the arterial “switch” operation (ASO) program in 2003. Until then, most of the babies were unconditionally sent to GOSH through the National Health Insurance. It is fair to say that there had been no mortality in the patients operated abroad compared to our poor results in the first couple of years, ASO being a procedure regarded everywhere as uniformly successful. The Norwood operation for hypoplastic left heart syndrome was started at the same time.

That was a difficult period with my occasional personal pessimistic plunges into the darkness. Nevertheless, my younger colleague who had also started operating the ASO and I had tremendous support from our cardiology colleagues.

Beside cardiac surgeons, the cardiology team is very engaged in international cooperation and education on multiple levels.

**Figure 2:**
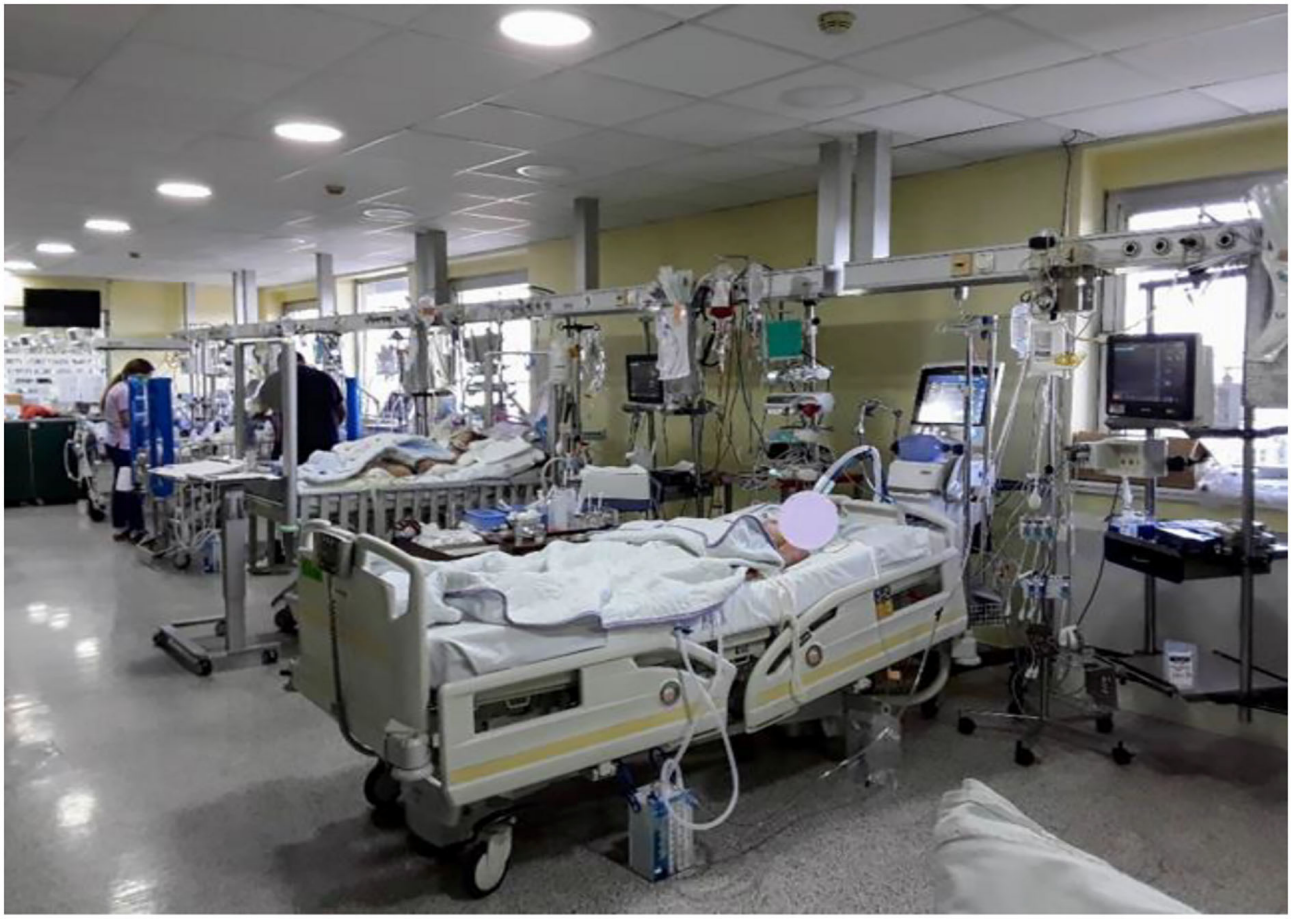
Cardiac catherizations at the MCHI in the period 2000–2020.

Our relationship went into another dimension when interventional cardiology started to flourish. There were only occasional surgical reinterventions, but the reality of a surgical solution to a cardiological complication brought us closer and increased empathy between us. In the current era, the younger and enthusiastic cardiologists are rightly expecting from us contemporary and complex surgery and hybrid procedures. With my experience from previous different circumstances, I tend to be on the cautious side. Luckily, my younger surgical colleague has the ambition to take this relationship to a new, higher level. The coronavirus pandemic has slowed down the interactions and training, thus complicating again our work (Figure 2).

#### Cardiac anesthesia and cardiac intensive care unit

**Figure 3:**
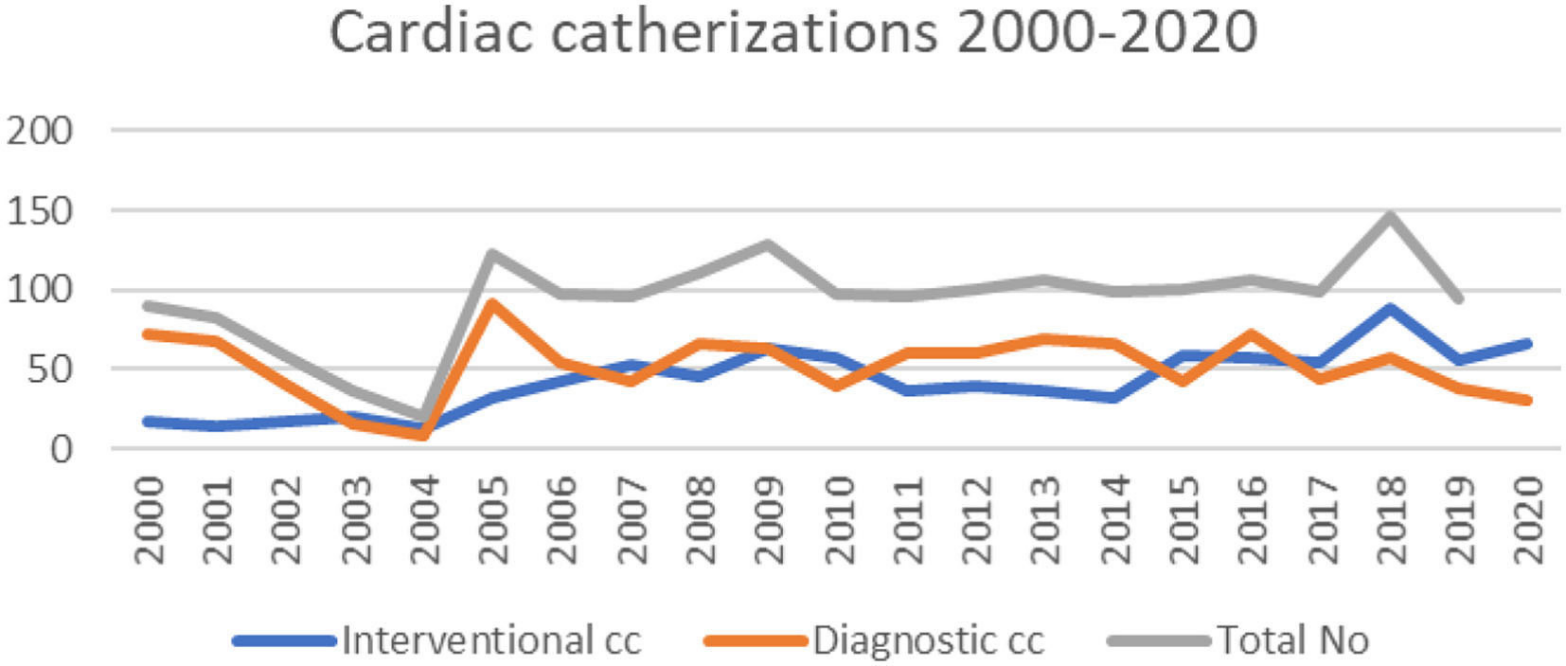
Anesthetic machine in 1997/1998.

In the beginning of our department, there were two qualified cardiac anesthesiologists. They were good physicians with completely different characters for the fact that greatly influenced, from my point of view, the recruitment and education of the new anesthesia doctors. The senior doctor was a cosmopolitan person, the pioneer of the modern pediatric anesthesia in our country. The second was an excellent, hardworking doctor, but always overshadowed by his senior colleague. His “overprotection” of younger colleagues and biased personal opinions have turned away a couple of young talented residents keen to be involved in our team. The next generation of anesthesiologists, two of them, in the late 80's, were exclusively dedicated to pediatric cardiac surgery, and my feeling is that good results in that era were influenced by their presence. Unfortunately, both of them left our hospital (Figure 3).

The constant deficiency of pediatric anesthesiologists in the region and worldwide had sent a lot of doctors abroad and made the remaining overpriced. Many anesthesiologists from lower graded institutions would fill up posts in our tertiary care hospital and after acquiring adequate skills would leave; for example, in 2002, we were left overnight without two cardiac anesthesiologists without notice. This pushed our program backward and led to another depression. Luckily, we recovered relatively quickly with the enormous enthusiasm of the rest of the team.

It took three or four generations of anesthesiologists within this 25-year period to dedicate to pediatric cardiac anesthesia in a contemporary and scientific manner. We are lucky now to have three hardworking and loyal doctors in whom I have the uttermost confidence. They work both in general pediatric and cardiac surgery.

The cardiac intensive care unit (CICU) has never been really led by anesthesiologists. The CICU is up to this day lead by surgeons. Although other specialties, like cardiologists, anesthesiologists, and pediatricians, are involved, and the cardiac surgeons make the final decisions. This is not the situation that I had wanted, but personal preferences of other specialists as well as objective circumstances never created an optimistic and competent intensivist to take the burden of the intensive care completely off our back. If we had to choose between doing the additional work in the CICU or having a “slow track” and old-fashioned approach to the postoperative care, my surgical colleagues and myself never had any doubts about rejecting the latter. The profile of an intensivist in our country was introduced as late as at the beginning of the 21st century, previously with opposition from both senior pediatricians and anesthesiologists. Nowadays, we have two intensivists of pediatric and cardiological profile. Although they participate in the CICU patient management with adequate theoretical knowledge of the surgical interventions, hemodynamics, postoperative care, and management, and neither of them are willing to do anything beyond the internistic scope, meaning that surgeons need to be involved in even the simplest surgical procedures, such as drain removal and placement of urinary catheters. Unfortunately, there is still no proper training for this valuable profile and is based on individual enthusiasm and engagement.

As we all know, one of the determining factors for the success of pediatric cardiac surgery operations is a meticulous preoperative assessment and management, especially in the neonatal age-group. The referred neonates are always admitted to the pediatric neonatal intensive care, where they are managed by pediatricians. I feel that there is still space for improvement on this level. There is no common cardiac intensive care unit managed by people who are familiar with complex CHD.

### Cardiac nurses, perfusionists, and nursing education

In the beginning of my cardiac surgery management, the choice of the CICU nurses was based only on previous skills and experience. No specialized courses or education was provided for CICU nursing staff. This had been a step back from the early history of our department when the most experienced nurses would accompany surgeons during international visits (mostly to Germany). The long-standing connections with The Hospital for Sick Children, Great Ormond Street Hospital in London, resulted in the organization of nursing and perfusionist fortnight or monthly visits. My personal feeling had always been that the nursing staff should not be deprived of international education and participation in conferences within possibilities.

Today, there are about 90% of nurses with specialized nursing secondary school and 10% with higher educational levels. The head nurses and the nurses in the organizational positions are required to have higher degrees of education. Continuous medical education is mandatory, but specialized education in CHD is mostly organized on a personalized level by doctors and nurses, but not as a required field. The principle of gradual training and nursing job grade classification does not exist in our country as a recognized method of professional advancing. The ascent into the CICU is based on personal skills, interest, and willingness to learn. Unfortunately, this scheme has been disrupted in the past couple of years because of massive emigration of nursing staff and perfusionists, mostly to the European Union (EU) or the Middle East. Thus, nowadays, it is not an uncommon situation that young and inexperienced nurses are pushed too early into the intensive care units due to the lack of staff. This situation requires additional energy and time to supervise them adequately by senior individuals. The low basic salary of the nursing staff and private workout of the hospital hours is also a distracting factor from CHD training.

The doctor–nurse interaction has been probably the most advanced improvement in our CICU compared to other intensive care units. This had started with the introduction of the ICHFNO and the “American” approach, which gave the nursing staff more freedom of action. It was not always greeted gladly both by individual doctors and nurses. The senior nurses felt insulted that their knowledge based on experience only was disturbed by younger staff acting out of their league, by having more theoretical knowledge that was reserved for doctors. With the gradual change of generations and with time, we have adopted the approach that nurses can act more independently, changing the ventilator parameters, inotrope dosage alterations, etc. as implemented by doctors.

**Figure 4:**
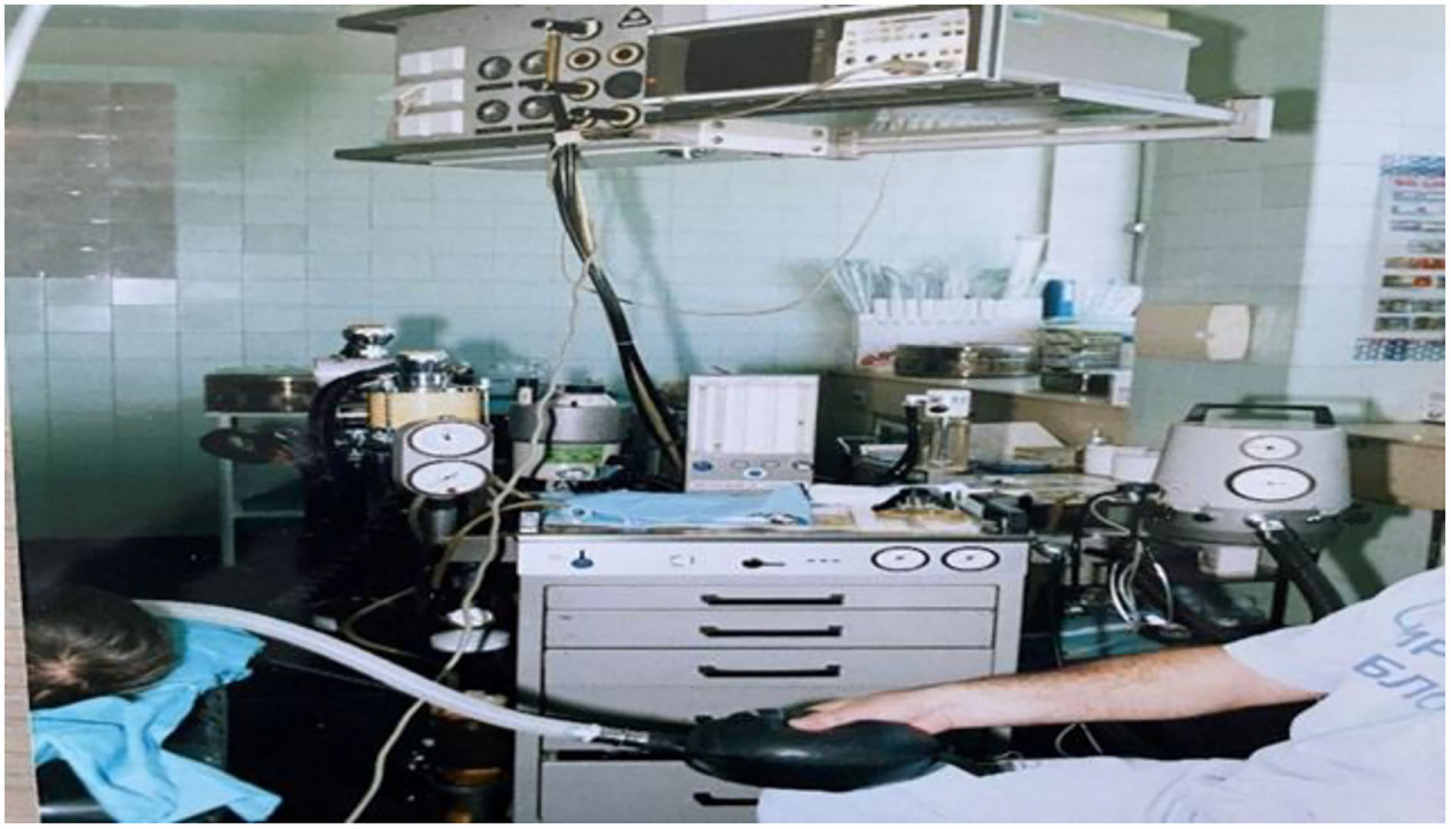
Operation Room in 2021.

The perfusion department now consists of three very different but dedicated individuals. The perfusionist, who had made the biggest impact on pediatric perfusion and was my “protégé” for nearly 15 years and who had started with me in my early surgical period, left the hospital 10 years ago. My professional relationship with him was frequently misunderstood because it took time for others to acknowledge the fact that pediatric cardiac perfusionist was not just a technician running the pump and that extracorporal circulation was a science on its own. Our current most senior perfusionist has international experience, and the two remaining do not, one for the language barrier, the other one for lack of opportunities. Our perfusionists are all active members of the Serbian Society for Extracorporeal Circulation and Technology (SrbSECT), which was founded in 2006 (8) (Figure 4).

The continuing challenge is commencement of the extracorporal membrane oxygenation (ECMO) program. Although we have the equipment and disposables, we have not started this procedure yet. The main problem is the lack of staff that would and could be dedicated to this technique. In practice, this would mean that we would have to cut down the elective and urgent operative list if we got engaged with ECMO. Knowing the fact that many patients needing ECMO are non-cardiac, this would mean involving pediatricians in the process, which would be a challenge on its own. The coronavirus pandemic is the other factor which had slowed us down. Arrangements were made for the doctors, perfusionists, and nurses to complete ECMO courses, but that has all been delayed in 2020.

### Stages of surgery and levels of excellence

From 1997 until the end of 2020, a total number of 3130 surgeries were performed, of which 2019 were open-heart procedures (64.5%). I do not have the formal results of the other center; my estimation is that our workload is more or less the same. During this period, our country changed from the Federal Republic of Yugoslavia to Serbia, and population decreased from 10.6 million in 2002 to 6.9 million in 2020. We accept referrals from other neighboring countries, mostly Bosnia and Herzegovina, Montenegro, and Macedonia.

…*It was March 1999. The political situation in Serbia (Yugoslavia) was cataclysmic. The ICHFNO was in Belgrade on a mission. Half of the team left Serbia a couple of days prior to NATO bombing. Dr. Novick and his closest associates stayed, and on the evening of March 23rd 1999, an arterial switch procedure was being done. I was assisting Dr Novick when the bombing started. As our operating theaters are on the 12th floor, we could see and hear the detonations in Batajnica, a village close to our hospital, where the military airport is situated. The Americans left via the land route the next day to neighboring Croatia…*

During the post-ICHFNO period, we had tried to follow the principles that we got accustomed to. In 2003, we started to operate on babies with hypoplastic left heart syndrome and D-transposition of great arteries (D-TGA; arterial switch operation, ASO). The results were unsatisfactory in the first couple of years not only due to my (in)experience with this operation, which I had started doing without senior supervision, but also due to the inexperience of others. I had presented our work with the ASO in my final subspecialty cardiac surgery exam, where I demonstrated that preoperative and postoperative management had a big impact on the survival (9). The results with the ASO improved and are good nowadays, even with complex forms. Our results with the Norwood operation are not satisfactory, even today. The results in the most challenging neonatal operations (interrupted aortic arch, total anomalous pulmonary venous return, coarctation of aorta, systemic to pulmonary shunts) are at a steady and acceptable level compared to larger centers.

We had always insisted on inviting colleagues from abroad to help with operations we had little or no experience with (complex left ventricular obstructions, the “double switch” procedure, Nikaidoh operation, etc.). My strong belief is that rare and complex procedures should be done in the country by someone who has more experience than us—there is no place for vanity. Since the departure of the ICHFNO, we have had individual visits of foreign surgeons from Italy, Greece, Turkey, the Great Britain, Germany, the United States, and Hungary. They performed operations on complex cases. My team enjoys having guests from abroad—it is a perfect way for learning and expanding horizons. Our National Health Insurance and the Ministry of Health support financially for sending children abroad for complex cardiac surgery. Beside the 89 children operated during the ICHFNO project, 44 children were operated by invited surgeons in the period 2001–2020. I am happy to say that the number of children needing this kind of help is decreasing every year (2).

The most recent professional communication on a department level is through the International Quality Improvement Collaborative (IQIC) (Boston, Mass., USA), which started in 2013 (10). This had a different impact on our department from the ICHFNO, under different circumstances, introducing the conception of quality control through an existing database. Unfortunately, due to the lack of financial support, we could not apply for memberships in other organizations and participate with data exchange.

The pediatric heart or heart/lung transplant service does not exist in this country. Serbia is not a part of the EU and as such not a part of Eurotransplant, which makes this form of treatment next to impossible. Adult cardiac transplantation has been functioning for a couple of years, and we had one patient of our hospital, adolescent, being successfully transplanted after multiple operations on the left ventricular outflow tract. Very rare pediatric patients have been transplanted abroad.

Although the legal cases in our country against medical malpractice are on the rise, we are still not officially obliged to present our results of surgical treatment CHD to the health authorities until this day. We have had no legal issues so far. I am very persistent that the preoperative surgical contact with the parents and the preoperative written consent should be very detailed and descriptive, quoting maybe even higher operative risks than in the Western world.

### Surgical results

Our department and pediatric services cover the age-group from the newborn age until the age of 18 (or final year at school). All cases above that age are transferred to the adult cardiac surgery service. Less complicated cases surviving to adulthood are operated by our colleagues, but more demanding grown up congenital heart (GUCH) patients are operated by visiting foreign specialists.

We had been obliged to write intrahospital annual reports from 2004, but with no retrospect on specific surgical issues. Since 2013, we joined the database of the International Quality Improvement Collaborative (IQIC) for Congenital Heart Surgery. As of today, our hospital is the only registered center from the former Yugoslavia region. The collaboration with the IQIC and Dr. Christopher Baird enlarged our operative repertoire, introducing the Ozaki procedure and other complex valvular operations. The results of our center (preoperative, operative, and postoperative outcomes) can be seen individualized and compared to the remaining 71 centers in the IQIC annual reports.

We also engage adult cardiac surgeons from local centers to help us with older adolescents requiring valve replacements or procedures more frequent in the adults. This cooperation has been working very well.

**Figure 5:**
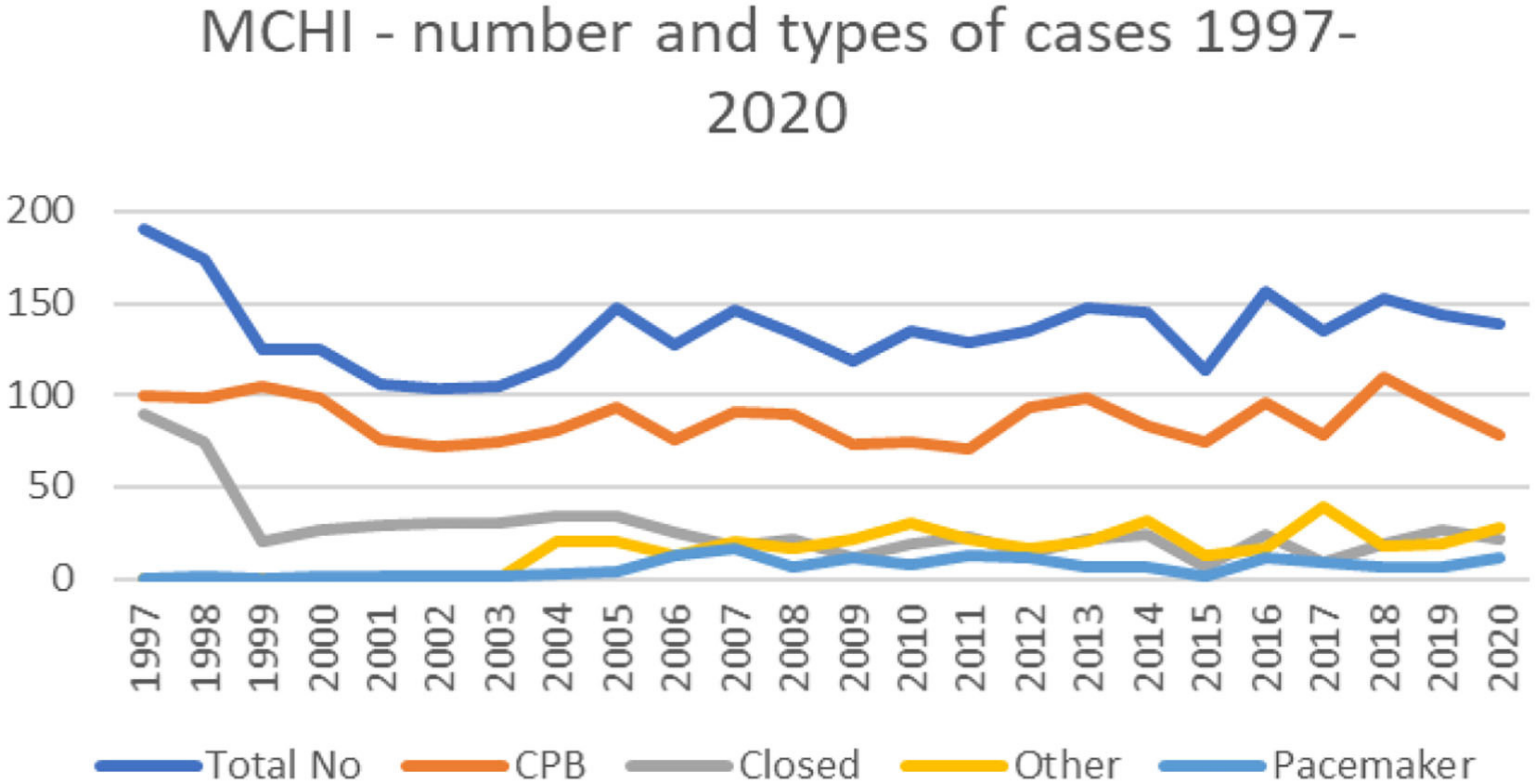
Number and types of surgeries 1997–2020.

**Figure 6:**
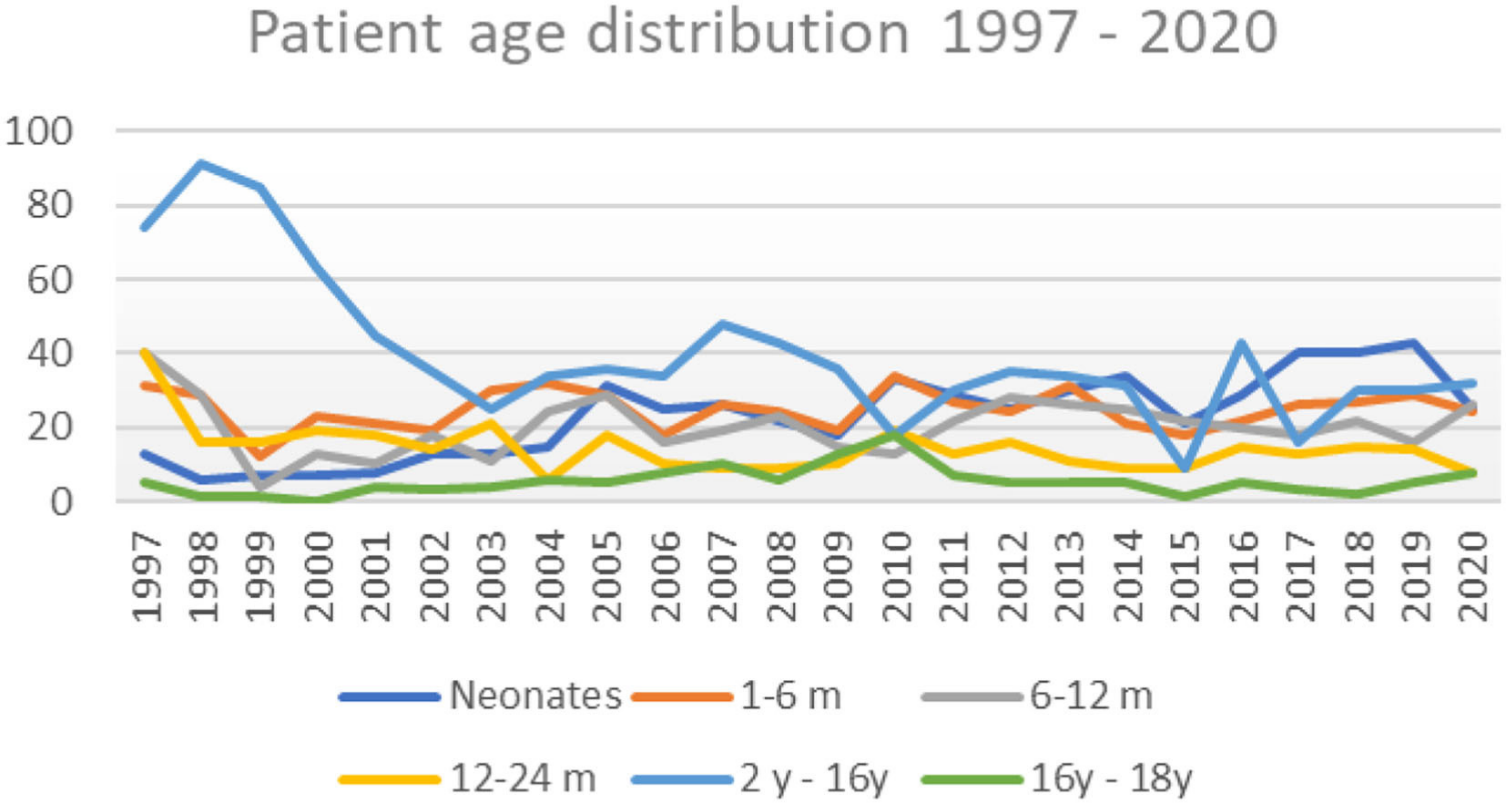
Patient age distribution 1997–2020.

It can clearly be seen that after the division of our department in 1998, the total case volume dropped and started to rise around 2004. The introduction of the Norwood operation and arterial switch operation also gave rise to the number of neonates operated annually (Figures 5, 6).

The number of newborns in this country fluctuates between 60,000 and 63,000 (Statistical Office of the Republic of Serbia), for instance, 63,484 newborns in 2019 and 61,693 in 2020 (11). The steady curve in the last 10 years shows, and presuming that the other center operates on a similar level, that we, in two centers, operate on about 300-320 children with CHD per year, which is sufficient to cover the surgical treatment of CHD according to the well-known and accepted incidence of 6–8% (12).

We are, thereby, presenting charts of basic distribution of our case volumes, patient age, mortality (since 1997), and Risk Adjustment for Congenital Heart Surgery (RACHS) distribution since 2008 (Table 1).

**Table 1:** RACHS distribution 2008–2020.

**RACHS**	**Sep 2008–Jun 2017 (%)**	**Jan–Jun 2017 (%)**	**2018 (%)**	**2019 (%)**	**2020 (%)**
1	17.8	12.5	14.2	8.4	8.1
2	40.7	43.8	39.4	48.7	34.3
3	28.1	29.2	34.7	29.4	39.4
4	6.6	8.3	5.5	9.2	15.2
5	0.2	0	0	0	0
6	2.3	4.2	5.5	4.2	3

**Figure 7:**
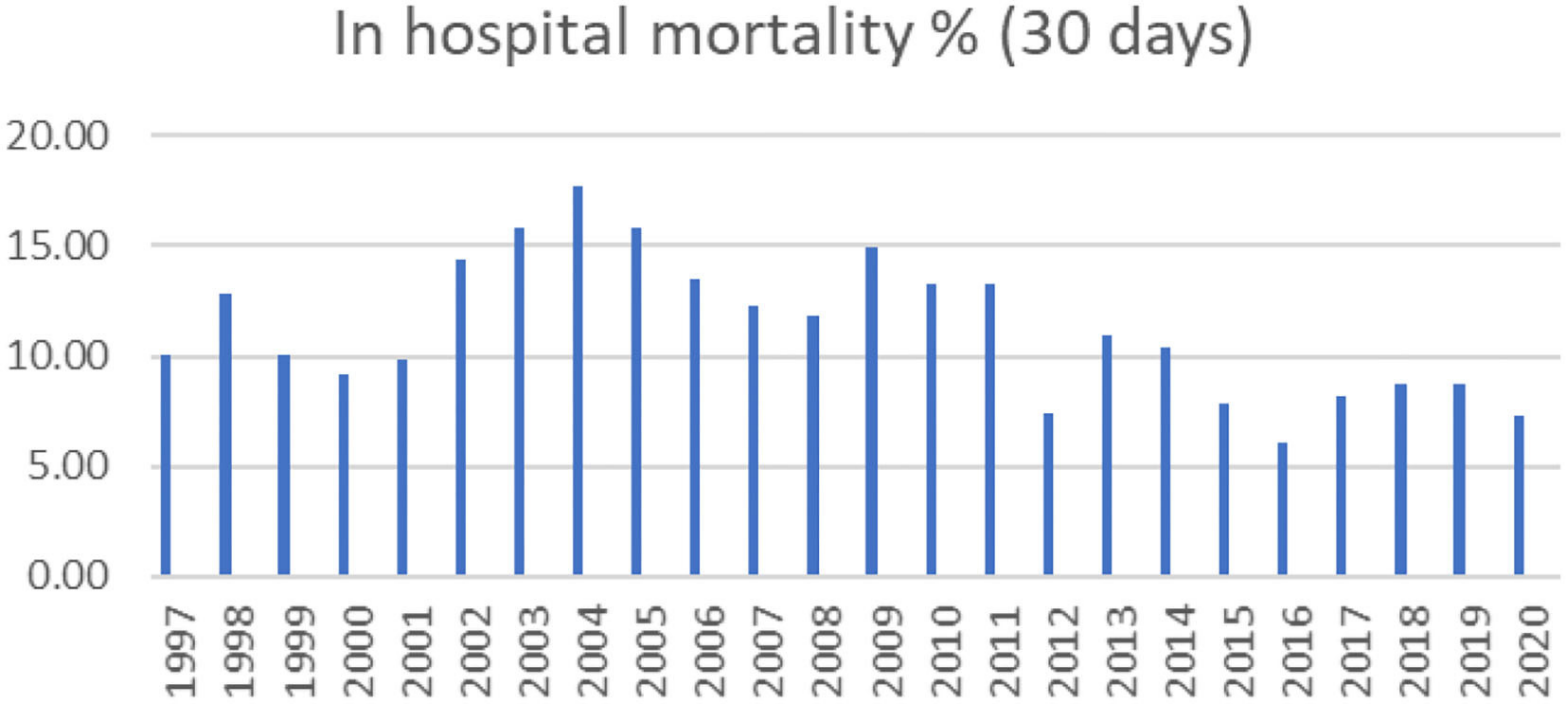
In-hospital 30-day mortality 1997–2020.

The mortality rates have always been calculated and are shown by year (Figure 7). The general increase in mortality in 2003 and 2004 can be attributed to the start of the ASO and Norwood procedures. Overall, 58% of our deceased patients are neonates. Our results with the hypoplastic left heart syndrome and the Norwood operation are unsatisfactory. They have been performed by three different surgeons and two foreign specialist with more or less the same outcome. In our yet uncompleted analysis of the risk factors concerning this condition, inadequate preoperative management of the babies seem to dominate.

## Position of pediatric cardiac surgery in Serbia today

### Funding and financing of pediatric cardiac surgery

**Figure 8:**
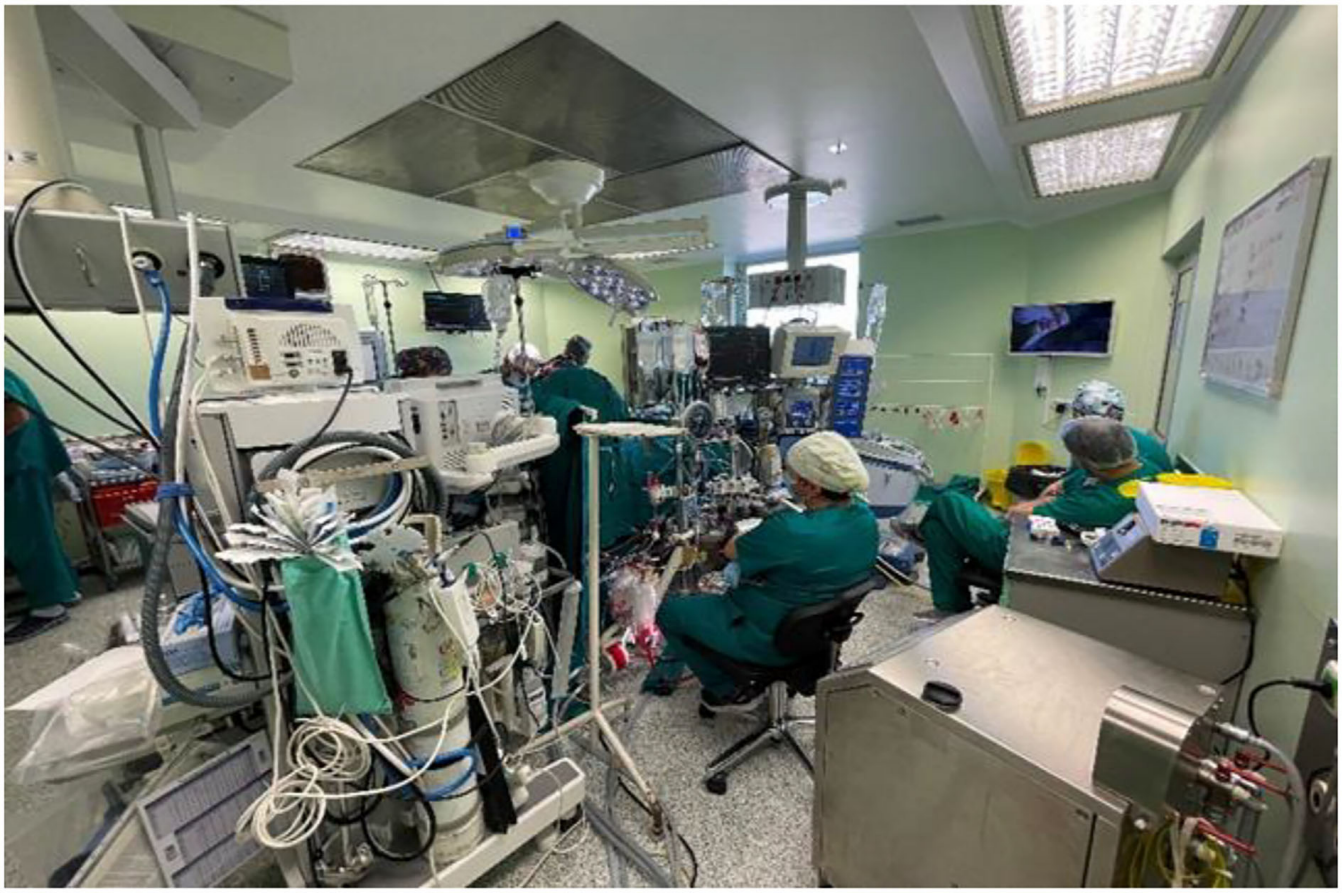
The CICU in 2021.

The pediatric cardiac surgery in Serbia is performed in two university state hospitals funded by the National Health Insurance; it has never been performed privately. After the founding of our department in 1982, occasional pediatric patients were operated in adult cardiac centers. One of the most difficult tasks we have had, in the pediatric specialty, was to fight through bureaucracy and separate the funding for pediatric supplies from adult financing. This brought to fruition within the last 15 years. The current status of our equipment is much better than the equipment 20 years ago. In the last 12 months, our hospital has been completely renewed with contemporary equipment financed by the European Union (Figure 8).

The National Health Insurance and the Ministry of Health have separate budgets for sending patients abroad in cases when we cannot offer promising and contemporary results. Paradoxically, these sums are annually larger than our institutional budgets. According to the unofficial data acquired from the National Health Insurance of Serbia and from my personal database (I am a member of the committee for sending patients abroad), about 60 patients were sent to foreign institutions for pediatric cardiac surgeries in the period from 2011 to 2019. The number of patients who were operated on by foreign teams or individual foreign cardiac surgeons in our premises, from 1997 to this day, was 133. The ICHFNO team operated on 89 children. The number of patients sent abroad for surgery is decreasing.

Our salaries are significantly lower and cannot be compared to the wages in the Western world. The introduction of salary band in the public sector and medicine has been delayed indefinitely. Cardiac surgery is in the same range as other specialities. Most of the surgeons take the in-hospital on-calls until retirement. The director and hospital administration can decide and provide additional the so-called financial “stimulations” up to 30% of the basic salary for teams, individuals on a permanent or single basis. The working hours in cardiac surgery are often beyond legally financed limits, and additional hours spent at the hospital are added up in the form of so-called “free days,” which are often three-digit numbers and cannot be “spent.” The younger generations of doctors often quote this poorly paid but demanding and complicated specialty as the turn-off point for joining.

### Surgical training, academic work, and publishing

Until 2008, our country did not have a separate cardiac surgery training program. Cardiac surgeons would qualify either as general or pediatric surgeons. From 2008, the medical school, University of Belgrade, established a 6-year postgraduate cardiac course with specialization in cardiac surgery (13). I had qualified in pediatric surgery in 1994 and in cardiac surgery in 2013. In order to officially qualify, a shorter, 4-year subspecialty course was granted to those to who have already practiced cardiac surgery independently for more than 10 years.

I had entered the medical school, University of Belgrade, in 1999 starting as an teaching assistant, hopefully claiming my full professorship in 2022. I teach both pediatric and cardiac surgery, pre- and postgraduate. We have courses for pediatricians, adult cardiologists, and neonatologists, which have helped the understanding of CHD in those specialty groups. Our main goal now is to help forming an adult congenital surgical team and find suitable young surgeons who will work on both sides, adult and congenital.

Publishing scientific articles from Serbia in the CHD field is not easy. We are a small population with a constant zero funding for science or experimental work. The hospitals do not have sources to finance the publishing fees. Still, a substantial number of articles emerged from our departments, based on huge personal enthusiasm and personal financing. Unfortunately, the shortage of qualified academic administrators and secretaries is considered a privilege we do not have in our department. The loss of time and energy that are wasted on administration and basic paperwork influences our work immensely.

### A woman in pediatric cardiac surgery

This is a topic I would have not mentioned in this article, but the ongoing sessions named Women in Cardio-Thoracic Surgery (WiCTS) at most recognized conferences in the last decade oblige me to do so. Until recently, I was the only female cardiac surgeon in Serbia, I believe even in the former Yugoslavia region. We have a couple of young ladies who have or will qualify shortly, but their preference is adult cardiac surgery. Although I admire most of my female surgeon colleagues of whatever specialty, I witness gossip blaming me to dislike women in cardiac surgery. This is not true. The truth is that working in Serbia, I am not a big fan of pointing up gender differences in our job in the 21st century in spite of the rising political and social awareness on the subject. You are either a good hardworking surgeon or not, and this applies for both genders. Our country differs somewhat from the centers in the USA and Western Europe. In the state-led hospitals where we work, there is no gender pay gap. On the other hand, the maternity leave policy is very liberal, allowing the woman to be away from work up to 2 years, which most, if not all, my female colleagues use. As far as motherhood and our profession go, there are undeniable facts that children and private life do not go easily with cardiac surgery, but they are possible with setting life priorities at the right time and being aware that certain sacrifices are bound to be made. I have experienced that all my professional life, being a single mother after a short-lasted marriage. For me, the more cruel reality in a relatively conservative and patriarchal communities such as Serbia are easy and arbitrarily made conclusions about female surgical capacities made by men, especially at the beginning of their careers. Not once have I been sneered and mocked by male colleagues. I am not ashamed to admit that I had shed tears and combined with the difficult profession in its own right, had many sleepless nights wondering if cardiac surgery was worth it. I am also not ashamed to admit that I fought back verbally whenever it was possible, but keeping the ultimate professional standards I could provide. This attitude toward women in cardiac surgery says more about the local community than the female individual cardiac surgeon. Changing the society in a low- or middle-developed country takes much more than a conference course. The very fact that I am still standing and still enjoying my job in Serbia should give a positive example to my younger female colleagues. I hope.

## Discussion

“*Behind every exquisite thing that existed, there was something tragic.”*

*Oscar Wilde*

Pediatric cardiac surgery is a surgical discipline that deals with the most exquisite and delicate patients and often associated with tragic outcomes due to the complexity of the patient status and environments where the children are operated. The term “excellence” quoted frequently in congenital heart surgery is usually applied to situations where complex surgical procedures, highly trained individuals, and perfectly organized medical systems create remarkable results in the survival and quality of life of the sickest of children. Unfortunately, very few of us are lucky to live in such surroundings. The contradictory feelings of frustration of having knowledge and not being able to apply it to the fullest extent and, on the other hand, the divine feeling of healing children determine our lifelong medical fate. The contribution of this article to congenital heart surgery should be in sharing our experiences and different levels of “excellence” in a country that is continuously on the thin line between exquisity and tragedy.

## Author contributions

The author confirms being the sole contributor of this work and has approved it for publication.

## Conflict of interest

The author declares that the research was conducted in the absence of any commercial or financial relationships that could be construed as a potential conflict of interest.

## Publisher's note

All claims expressed in this article are solely those of the authors and do not necessarily represent those of their affiliated organizations, or those of the publisher, the editors and the reviewers. Any product that may be evaluated in this article, or claim that may be made by its manufacturer, is not guaranteed or endorsed by the publisher.
